# Histochemical Localization and Cytotoxic Potential of Alkaloids in *Phaedranassa lehmannii*

**DOI:** 10.3390/plants13223251

**Published:** 2024-11-20

**Authors:** Lina M. Trujillo Chacón, Hawer Leiva, José M. Rojas, Isabel C. Zapata Vahos, Dagoberto Castro, María Domínguez, Edison Osorio

**Affiliations:** 1 Grupo de Investigación en Sustancias Bioactivas GISB, Facultad de Ciencias Farmacéuticas y Alimentarias, Universidad de Antioquia, Calle 70 No. 52-21, Medellín 050010, Colombia; linam.trujillo@udea.edu.co; 2 Unidad de Biotecnología Vegetal, Facultad de Ingeniería, Universidad Católica de Oriente, Rionegro 054040, Colombia; yleiva@uco.edu.co (H.L.); investigacion.dir@uco.edu.co (D.C.); mdominguez@uco.edu.co (M.D.); 3 Grupo de Investigación Estudios Florísticos, Facultad de Ingeniería, Universidad Católica de Oriente, Apartado Aéreo 008, Rionegro 054040, Colombia; jose.rojas6749@uco.net.co; 4 Grupo de Investigación Atención Primaria en Salud, Facultad de Ciencias de la Salud, Universidad Católica de Oriente, Rionegro 054040, Colombia; izapata@uco.edu.co

**Keywords:** *Phaedranassa lehmannii*, Amaryllidaceae alkaloids, cytotoxicity, histochemical localization

## Abstract

Plants of the subfamily Amaryllidoideae are a source of unique and bioactive alkaloids called Amaryllidaceae alkaloids. The study of their anticancer potential has intensified in recent years. This work aims to locate and characterize the profile of cytotoxic alkaloids biosynthesized and stored in different tissues of *Phaedranassa lehmannii* Regel using different histochemical methods and chromatographic analysis. The histochemical analysis in the bulbs revealed the presence of alkaloids at the basal edge of the scale-like leaves and bud apical zone. The GC-MS analysis indicated that the bulbs biosynthesize crinane- (9.80 µg/g DW), galanthamine- (8.04 µg/g DW), lycorine- (7.38 µg/g DW), and narciclasine-type (3.75 µg/g DW) alkaloids. The root biosynthesizes alkaloids that are mainly distributed mostly in lycorine- (225.29 µg/g DW) and galanthamine-type (72.35 µg/g DW) alkaloids. The total alkaloids biosynthesized by the root (324.93 µg/g DW) exceeded eleven times the abundance of the alkaloids identified in the bulbs (28.97 µg/g DW). In addition, the total alkaloid fractions exhibited a dose-dependent cytotoxic effect in the evaluated concentrations, with IC_50_ values of 11.76 ± 0.99 µg/mL and 2.59 ± 0.56 µg/mL against human lung (A549) cancer cells and 8.00 ± 1.35 µg/mL and 18.74 ± 1.99 µg/mL against gastric (AGS) cancer cells. The present study provided evidence to locate and characterize the alkaloids of *P. lehmannii* grown under nursery conditions as a species producing potential antiproliferative alkaloids.

## 1. Introduction

The family Amaryllidaceae is a monophyletic group represented by approximately 1650 species distributed in 73 genera. Three clades are recognized, represented in the subfamilies Agapanthoideae, Allioideae, and Amaryllidoideae. The subfamily Amaryllidoideae is made up of monocotyledonous, bulbous, and flowering species [1]. The subfamily Amaryllidoideae has increased its level of importance due to the presence of unique isoquinoline alkaloids biogenetically derived from tyrosine and phenylalanine and known as Amaryllidaceae alkaloids [2,3]. These alkaloids are characterized by their biological effects, such as neuroprotective, enzyme inhibitors of cholinesterase and butyrylcholinesterase, anti-inflammatory, and antiviral, among others [2]. Galanthamine, for example, is a potent inhibitor of cholinesterase and has demonstrated a superior response in clinical terms in comparison with other Amaryllidaceae alkaloids [4]. For this reason, it has been approved for the treatment of Alzheimer’s disease [5]. On the other hand, Amaryllidaceae alkaloids have been isolated and tested as antiproliferative agents for different types of cancer [6,7]. The biological potential shows that Amaryllidaceae alkaloids could be used in the pharmacological field of cancer [1,8].

In Colombia, 18 genera and 48 species of the Amaryllidoideae subfamily have been reported, of which 11 genera and 27 species are native, with 9 endemic species [9]. *Phaedranassa*, one of these genera, corresponds to perennial herbs, up to 60 cm tall, with bulbs 2–6 cm in diameter, globose, with cream to brown tunic. The species of the genus are distributed in tropical and premontane forests of the southern Cordillera Occidental and Nudo de los Pastos, reaching the center of Ecuador [9]. Although phytochemical analysis of *Phaedranassa* has shown three different Amaryllidaceae alkaloid types (lycorine, crinine/haemanthamine, and galanthamine), the alkaloids identified vary according to the species [10,11]. *Phaedranassa cinerea*, *Phaedranassa cuencana*, and *Phaedranassa ventricosa* are characterized by producing lycorine- and galanthamine-type alkaloids [7,10]. In *Phaedranassa brevifolia*, *Phaedranassa dubia*, *Phaedranassa glauciflora*, and *Phaedranassa tunguraguae*, the presence of crinine/haemanthamine-, lycorine-, and galanthamine-type alkaloids is notable [10,11,12]. In addition, alkaloids, such as crinine-, lycorine-, and narcyclasine-type, have been reported in *Phaedranassa lehmannii* [13,14]. Other less frequent alkaloid types are homolycorine and montanine, found in fewer species [10,12]. In this way, the production of crinine- and lycorine-type alkaloids in *Phaedranassa* species is highlighted.

In the search for new molecules for the treatment and prevention of cancer, the vegetative organs of many Amaryllidoideae species have been analyzed as a source of numerous cytotoxic Amaryllidaceae alkaloids, particularly crinane- and lycorine-type alkaloids [15,16]. A correlation has been suggested between the cytotoxic effect of Amaryllidaceae species and crinane- and lycorine-type alkaloids [7]. Simultaneously, the location, composition of the chemical profile, and distribution of alkaloids in producing organs are considered important productive features of the subfamily Amaryllidoideae due to the number of compounds in low concentrations [2]. This could be a strategy to increase the availability of cytotoxic alkaloids with interesting pharmacological activities, which are limited in nature by plants of the Amaryllidoideae subfamily. Histochemistry identification may yield valuable data on the producing organs and cell-type localization of the Amaryllidaceae alkaloids [17]. For this, different histochemical methods have been developed, which allow the characterization of plant structures, where different compounds are distributed and accumulate, as well as the precise determination of tissues involved in the secretion of alkaloids [18]. However, studies of spatial distribution, localization, and accumulation of Amaryllidaceae alkaloids in different plant tissues (bulb, leaf, and root) are limited, especially in cytotoxic Amaryllidaceae alkaloids. In most cases, and for industrial purposes, histochemical studies have been performed on the main sources of galanthamine for further optimization of compound production [17,19]. Therefore, this work aims to locate and characterize the alkaloids in the underground organs (bulbs and roots) of *P. lehmannii*, a species with interesting pharmacological properties and a source of cytotoxic alkaloids [7]. For this purpose, histochemical methods and chromatographic analysis were used on plants grown under nursery conditions.

## 2. Results

### 2.1. Findings in Histochemical Analysis

**Figure 1:**
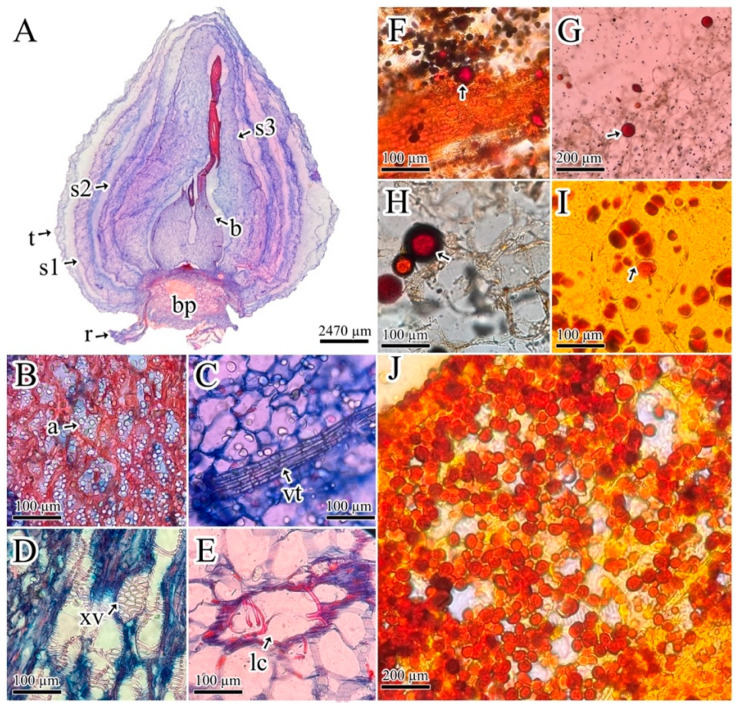
*Phaedranassa lehmannii* bulbs anatomy. (**A**) Anatomic details of bulb tissues. (**B**) Amyloplast storage in the basal plate; (**C**) bud developing vascular tissue; (**D**) xylem vessels; (**E**) lysogenic cavities; (**F**–**J**) detection of alkaloids in the bud and scale leaves; (**F**,**G**) Wagner’s reagent; (**H**) Lugol’s reagent; (**I**,**J**) Dragendorff’s reagent. Arrow shows alkaloid storage: a = amyloplast, b = bud, bp = basal plate, lc = lysogenic cavities, r = root, s1 = scale leaf 1, s2 = scale leaf 2, s3 = scale leaf 3, t = tunic, vt = vascular tissue, xv = xylem vessels.

*P. lehmannii* bulbs develop a thin tunic and three scale-like leaves that protect the bud (Figure 1A). Scale leaves are made up of thin-walled polyhedric parenchyma cells. The scaly leaf closest to the bud develops lysogenic cavities in the apical zone (Figure 1E). In addition, the bulbs present a basal plate formed by vascular tissue (xylem vessels) and parenchyma flattened cells, which have amyloplast storage (Figure 1B,D) and a central bud provided by a base formed by parenchyma-like storage cells, while the bud apical zone has developing vascular tissue (Figure 1C). Dragendorff’s and Wagner’s reagents, with reddish-brown staining, and Lugol’s reagent, showed alkaloid storage at the basal edge of the scale-like leaves and the bud apical zone, with the absence of alkaloids on the basal plate (Figure 1F–J).

### 2.2. Chromatographic Analysis of Alkaloids from P. lehmannii

**Figure 2:**
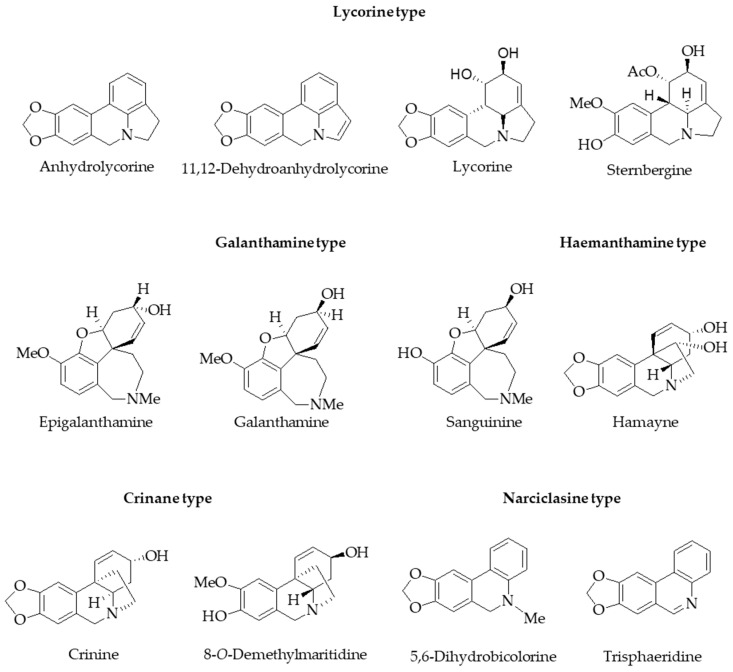
Chemical structures of alkaloids identified in bulb and root from *Phaedranassa lehmannii*.

After histochemical and phytochemical detection with different staining reagents, the alkaloids present in the bulb and root of *P. lehmannii* were analyzed by GC-MS. In the total alkaloid fraction of bulbs, eight alkaloids were detected with a predominant abundance of crinane- (9.80 µg/g DW), followed by galanthamine- (8.04 µg/g DW) and lycorine-type (7.38 µg/g DW) alkaloids (Table 1; Figure 2). The alkaloids 8-*O*-demethylmaritidine (8.77 µg/g DW) and lycorine (6.79 µg/g DW) were the most abundant. Interestingly, the root showed biosynthesis of nine alkaloids with high diversity and abundance. Lycorine-type alkaloids (225.29 µg/g DW) predominated in the root, with a high prevalence of anhydrolycorine (133.7 µg/g DW) and lycorine (73.97 µg/g DW) being the most abundant compounds in the total alkaloid fraction, followed by galanthamine-type (72.35 µg/g DW) and narciclasine-type (13.74 µg/g DW) alkaloids. Additionally, the results showed that the alkaloids biosynthesized by the root (324.93 µg/g DW) exceeded eleven times the abundance of the alkaloids identified in the bulbs (28.97 µg/g DW).

### 2.3. Cytotoxic Activity from P. lehmannii Alkaloids

**Figure 3:**
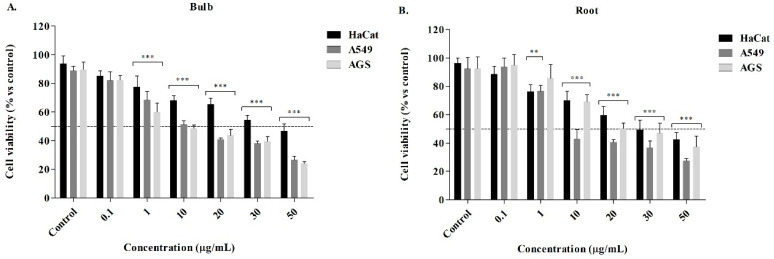
Cell viability of alkaloid fractions of the bulb (**A**) and root (**B**) in gastric AGS and lung A549 cancer cells. Human keratinocytes HaCat were used as control cells. The results correspond to the mean ± SEM (n = 3). ** *p* ≤ 0.01 and *** *p* ≤ 0.001 indicates statistically significant differences between control (DMSO 0.1%) and treatments according to Dunnett’s Multiple Comparison Test.

The alkaloid fractions of bulbs and roots showed a cytotoxic effect in the evaluated concentrations with IC_50_ values of 11.76 ± 0.99 µg/mL and 2.59 ± 0.56 µg/mL against human lung (A549) cancer cells and 8.00 ± 1.35 µg/mL and 18.74 ± 1.99 µg/mL against gastric (AGS) cancer cells over 48 h exposure, determined by the reduction of 3-(4,5-dimethylthiazol-2) 2,5-diphenyltetrazolium bromide (MTT) to formazan. The alkaloids showed a concentration-dependent decrease in cell viability with statistically significant differences for concentrations greater than 1 μg/mL (Figure 3A,B). Lycorine and doxorubicin were tested as standards (positive control), with IC_50_ values of 4.97 ± 0.89 µg/mL and 5.52 ± 0.21 µg/mL, respectively (Table 2). The values showed that alkaloids biosynthesized in the root generate a high cytotoxic effect on lung cancer cells compared with lycorine. Alkaloid fractions were also evaluated on human keratinocytes (HaCat, non-cancer control cells) to determine the degree of alkaloid selectivity by determination of the selectivity index (SI, ratio of IC_50_). In general, the alkaloid fractions showed that concentrations ranging from 0.1 to 50 µg/mL resulted in 44–88% cell viability in HaCaT cells, with no significant differences at low doses. SI values higher than 1.0 indicate that compounds or extracts have anticancer specificity. If SI values are much greater than 1.0, the samples are highly selective [20]. Based on this, the SI data shown in Table 2 indicate that alkaloids of bulbs, root, and lycorine standard exhibit a high degree of cytotoxic selectivity against lung and gastric cancer cells with values between 2.11 and 15.29.

## 3. Discussion

Alkaloids are molecules that plants use for chemical defense. Most plants produce alkaloids in one organ, then transport them through the xylem or phloem and store them in organs such as seeds, flowers, leaves, or more specialized storage organs such as bulbs [21]. In addition, it is well known that the production of metabolites depends on conditions such as the state of development of the plant and environmental conditions such as climate, location, and method of cultivation [22]. Specifically, in the genus *Phaedranassa*, a chemical diversity in the production of Amaryllidaceae alkaloid has been demonstrated [10,11,12,13,14], and it could be suggested that the production is regulated by the processes of alkaloid biosynthesis in specific tissues or cells, depending largely on the level of differentiation and development, and then stored mainly in bulbs and roots, as occurs in other species of the subfamily [17]. However, few studies have examined the anatomy and histochemistry of *Phaedranassa* bulbs. In most cases, histochemical studies have been performed on the main sources of galanthamine for the further optimization of compound production [17,19].

In Amaryllidoideae species, a previous study to identify the alkaloids produced by *Rhodophiala bifida* highlights alkaloid concentrations in bulbs and roots [3]. In *Narcissus pseudonarcissus,* high alkaloid (galanthamine) storage is reported in the basal plate, followed by the bulb tissues [23]. Research on *Zephyranthes irwiniana* bulbs identified alkaloids in the chlorenchyma cells of the leaf and bulb margins and reported that data on the histolocalization of Amaryllidaceae alkaloids are scarce [24]. However, our results show that alkaloid storage in *P. lehmannii* occurs both in the roots and bulbs, in the latter, in the scale-like leaves, and in the apical zone of the buds, but not in the basal plate (Figure 1). This confirms the possible movement of alkaloids between tissues, as suggested in Amaryllidoideae species [25]. Nevertheless, further studies are required to characterize the transport of cytotoxic alkaloids and identify in which tissues their synthesis and storage occur. In this regard, it has been shown in *Hippeastrum papilio* that galantamine biosynthesis occurs in green tissues and is transferred to other plant organs, for example, bulbs and roots [17].

The localization of alkaloids in plant tissues could provide a better understanding of the role and sites of biosynthesis and accumulation of these secondary metabolites. In addition, this contributes to the search for new sources and to the establishment of organogenesis processes aimed at the production of alkaloid-rich biomass without endangering the biodiversity of wild populations [26,27]. This strategy could increase the availability of cytotoxic alkaloids with interesting pharmacological activities, such as lycorine, which are limited by nature in plants of the Amaryllidoideae subfamily [28,29].

In the identification of alkaloids in *P. lehmannii* bulbs and roots, it was established that the alkaloids detected by CG-MS would be directly correlated with the positive staining accumulated in the central part of the root, the basal edge of scale-like leaves, and the bud apical zone. The fraction of total alkaloids of bulbs corresponded to a relative abundance of 28.97 µg/g DW with a high prevalence of crinane-, galanthamine-, and lycorine-type alkaloids, with an outstanding abundance of 8-*O*-demethylmaritidine, galanthamine, sanguinine, and lycorine (Table 1). The production of alkaloids in the root (324.93 µg/g DW) was eleven times higher than that reported for bulbs. In the root, the lycorine-type alkaloids were predominant, mainly anhydrolycorine and lycorine, followed by galantamine-type alkaloids, with a predominance of galantamine. This shows that the biosynthesis and accumulation of specific alkaloids can be limited to specific cell types, tissues, or organs [30,31].

This effect has been observed in Amaryllidoideae species, such as *Lycoris radiata*, where leaves and roots contain significantly more alkaloids of interest than in scapes and bulbs [31]. In *Leucojum aestivum*, the biosynthesis and storage of galantamine are higher in the bulb (0.0949 mg/g) than in the root (0.0262 mg/g), while lycorine is higher in the root (0.2328 mg/g) than bulb (0.1994 mg/g) [29]. Similarly, *Galanthus nivalis* produces twenty-two times more lycorine in the root (0.1816 mg/g) than in the bulb (0.0080 mg/g) [29,32]. Therefore, the results found for *P. lehmannii* are related to those reported for other plants of the subfamily Amarylllidoideae regarding the abundance of alkaloids such as lycorine and galanthamine. Interestingly, these two alkaloids are widely required by the pharmaceutical industry due to insufficient availability of natural resources and the significant increase in the demand for galanthamine for the treatment of neurodegenerative diseases [28] and the biological interest of lycorine for the search for therapeutic alternatives in cancer [33,34].

Although the alkaloids identified in *P. lehmannii* have been reported in previous studies [7,8,13,14], differences in the concentration of alkaloids and the presence of others previously not reported, such as sternbergine, were evidenced. This is possibly due to a change in some biological, chemical, or environmental factors that influence the biosynthesis and accumulation of secondary metabolites [35]. Concerning other species of the genus, 70% of the alkaloids identified in *P. dubia* and *P. brevifolia* correspond to lycorine- and haemanthamine/crinine-type alkaloids [12]. In *P. cinerea*, *P. cuencana*, *P. dubia*, *P. glauciflora*, and *P. tunguraguae*, approximately 50% of the alkaloids correspond to the lycorine-type and 15% to the crinine/hemanthamine-type alkaloids [10]. Therefore, plants of the genus Phaedrannassa are chemically interesting for their alkaloid profiles and pharmacologically of interest due to the cytotoxic potential that many of these alkaloids present [12].

The alkaloid fractions from *P. lehmannii* were evaluated in gastric (AGS) and lung (A549) cancer cells using lycorine as the reference alkaloid. The alkaloid fraction of the root showed a high cytotoxic effect on lung cancer cells with IC_50_ values of 2.59 ± 0.56 µg/mL, compared to the alkaloid fraction of the bulbs. The antiproliferative effect against lung cancer cells could be attributed to the high presence of lycorine-type alkaloids in the root (220.51 µg/g DW, with a relative abundance of 67.86%) every time that lycorine induces high cell death with IC_50_ of 4.97 ± 0.89 µg/mL, with a high degree of selectivity. In gastric cancer cells, the alkaloid fraction of bulbs (IC_50_ of 8.00 ± 1.35 μg/mL) showed the greatest decrease in cell viability compared to the root (IC_50_ of 18.74 ± 1.99 μg/mL). Consequently, the cytotoxic potential against lung and gastric cancer cells of the alkaloid fractions could be attributed to the presence of lycorine-, crinine-, haemanthamine-, and narciclasine-type alkaloids.

It has previously been reported that lycorine-type alkaloids and lycorine have cytotoxic efficacy against many cancer cell lines, including lung cancer [34,36,37,38]. Lycorine and related structural compounds have been shown to decrease proliferation, invasion, and metastasis in different lung cancer models [39]. In addition, lycorine is a potent inducer of apoptosis, both in mitochondrial and death receptor-mediated apoptosis. This mode of action is related to the inhibition of migration and proliferation of cells with resistance to apoptosis [36]. Although multiple apoptotic mechanisms of lycorine are possible, depending on the type of cancer, in human lung cancer cells, lycorine causes a G0/G1 phase arrest, induces an early stage of apoptosis, and initiates mitochondrial dysfunction [38]. Therefore, lycorine has been suggested as an excellent candidate for combating cancers [34,36], and new lycorine derivatives have been synthesized to verify the antiproliferative effects against different cancer cell lines [40].

The crinine-type alkaloids have also demonstrated antiproliferative properties in several cancer cell lines [41]. Haemanthamine- and narciclasine-type alkaloids have also received considerable attention for their potential antitumor properties [1,42]. Therefore, the cytotoxic potential of the alkaloids fraction of bulbs could be associated with the presence of lycorine-, crinine-, and narciclasine-type alkaloids, with relative abundances of 23.44%, 33.83%, and 12.95%, while for the root, the activity would be associated with lycorine-, haemanthamine-, and narciclasine-type alkaloids, with abundances of 67.86%, 4.23%, and 4.17% (Table 1). In addition, the results showed that the alkaloids lycorine (23.44%), 8-*O*-demethylmaritidine (30.27%), and trisphaeridine (12.95%) represent a high percentage in the total alkaloid fractions of bulbs. The alkaloids anhydrolycorine (41.15%), 11,12-dehydroanhydrolycorine (3.95%), lycorine (22.76%), hamayne (4.17%), trisphaeridine (3.49%), belonging to the previously mentioned types of alkaloids, are highly predominant in the alkaloid fractions of roots. Consequently, the structural diversity of the alkaloids present in bulbs and roots could condition the bioactivity response observed in the in vitro cancer model and, therefore, explain the variations in the results.

## 4. Materials and Methods

### 4.1. Plant Material

The plant material grown under greenhouse conditions was obtained from the Plant Biotechnology Unit, Faculty of Engineering, Universidad Católica de Oriente, Rionegro, Colombia. One specimen of the species was deposited in the Herbarium of the University of Antioquia, Medellín, Colombia (voucher 5106). The material studied was collected with authorization from the Ministry of Environment with a genetic resource access contract #328. The selection criteria were material not in bloom and not more than 20 cm in length.

### 4.2. Histochemical Analysis

Bulbs of *P. lehmannii* were fixated in formaldehyde, acetic acid, and ethanol (FAA) for 24 h at 6 °C [43]. After, each tissue was dehydrated in an ethanol series (50, 60, 70, 80, 90, 96, and 100%) and rinsed in HistoChoice^®^ (Amresco Inc., Solon Ohio, OH, USA) twice for 12 h. Subsequently, the tissues were embedded in a solution 2:1 composed of HistoChoice^®^ (Amresco Inc., Solon Ohio, OH, USA) and Paraplast^®^ paraffin (Amresco Inc., Solon Ohio, OH, USA) for 2 h and then in a 1:2 solution. Finally, the tissues were embedded in 100% Paraplast^®^ paraffin for 12 h three times [44]. Sections were made in different cutting planes of 5 and 7 µm thick, using a Leica RM2125 rotatory microtome (Leica Biosystems, Deer Park, TX, USA). Staining was done with Safranin-Alcian Blue (Fasga) for structural differentiation of tissues [45]. Dragendorff and Wagner methods were used for the detection of alkaloids [46]. With Dragendorff reagents, the alkaloids were stained red or red-brown. In the Wagner assay, the reagent gives colors similar to Dragendorff in the presence of alkaloids. Lugol’s reagent, which is similar in chemical composition to Wagner’s reagent, produces a golden-brown reaction [46,47,48]. Sections were observed through an Olympus CX31 optical microscope (Olympus Life Science, Waltham, MA, USA).

### 4.3. Extraction of Alkaloids

The treatment of plant material and extraction of alkaloids was carried out following protocols described previously [7,49]. The plants grown under greenhouse conditions were collected and washed with drinking water. After that, the bulbs were cut into pieces of 3 cm and dried at 40 °C for 48 h. The extract in methanol was made with dried and ground plant material, applying 15 min of ultrasonic baths (3 times), changing the solvent, and evaporating the solvent at reduced pressure. The concentrated extract was dissolved in 10 mL 2% H_2_SO_4_, hexane (3 × 10 mL) was used to remove neutral compounds, and ammonium hydroxide was added to the aqueous fraction to adjust pH in the range of 9 to 10. Then, the alkaloids were extracted with chloroform (3 × 50 mL). The organic solvent was evaporated using a rotary evaporator. To finish, for analysis in GC-MS, 5 mg of extract was dissolved in 500 μL methanol.

### 4.4. Chromatographic Analysis of Alkaloids

The analysis of alkaloids of *P. lehmannii* by GC-MS was performed according to the method described previously [50]. The alkaloid fraction was injected into an Agilent 7890 Gas Chromatograph (Agilent, Santa Clara, CA, USA) equipped with a 5975C selective mass detector and electronic impact (EI) operating at 70 eV in splitless mode, programmed to acquire signals in scan mode between 40 and 400 daltons. In an HP-1 MS capillary column (30 m × 0.25 mm × 0.25 μm), the alkaloids were separated with a carrier gas flow (Helium) of 1 mL/min. The temperature ramp was as follows: 100–180 °C at 15 °C/min, 180–300 °C at 5 °C/min, and 10 min hot at 300 °C. The temperature of the injector was kept at 250 °C. Subsequently, 1 μL of the sample was injected.

### 4.5. Determination of the Alkaloid Profile

To identify alkaloids, the database “Amaryllidaceae Alkaloid Spectroteca, Agro Bio Institute (Bulgaria)” and data reported in scientific journals were used to compare the fragments of the mass spectra of each molecule. The Kovats retention rates of the compounds were recorded with a standard calibration mixture of n-hydrocarbons (C7–C40). The percentage TIC (total ion current) was determined for each alkaloid. Codeine (50 μg/mL) was used as an internal standard, and with this, the abundance of each compound was calculated. The proportion of each compound in the extracts tested was expressed as a percentage of the total alkaloid content in the area of the GC-MS peaks depending on the concentration of the related compound and the intensity of their mass spectral fragmentation.

### 4.6. Cell Viability of Alkaloid Fraction

Cell culture: Cell viability of the total alkaloid fraction was evaluated in the gastric (AGS, CRL-1739™) and lung (A549, CCL-185™) cancer cell lines. Human keratinocytes (HaCat, PCS-200-011™) were used as a non-carcinogenic control cell line. Cell lines were obtained from the American Type Culture Collection (ATCC). The cells A549 and HaCat were grown cultured in Dulbecco Modified Eagle Medium-high glucose (DMEM) and AGS in Ham’s F-12 nutrient medium (F12) supplemented with 10% heat-inactivated FBS, 100 U/mL penicillin and 100 μg/mL streptomycin, 2 mM L-glutamine in a humidified atmosphere of 5% CO_2_ and 95% air at 37 °C. Cells were monitored on a Nikon Eclipse TS100 inverted phase contrast microscope (Marshall Scientific, Hampton, VA, USA). Cell viability experiments were performed when cells reached 75–80% confluence using 0.25% Trypsin 1 mM EDTA.

Cell viability by mitochondrial MTT reduction: Cell viability was determined using 3-(4,5-dimethylthiazol-2-yl)-2,5-diphenyltetrazolium bromide (MTT) assay. The total alkaloid fractions and lycorine standard were evaluated at concentrations of 50, 30, 20, 10, 1, 0.1 μg/mL and doxorubicin at 10, 5, 2.50, 1.25, 0.62, 0.31 μg/mL. Both the total alkaloid fractions and lycorine standard were prepared in dimethyl sulfoxide (DMSO). The final DMSO concentration in the medium did not exceed 0.1% *v*/*v* (volume/volume). All samples were prepared in dimethyl sulfoxide (DMSO). Cells were seeded in 96-well plates with a cell density of 2 × 10^6^ cells/mL and incubated for 24 h at 37 °C with 5% CO_2_. After this time, cells were incubated with samples at 37 °C with 5% CO_2_ for 48 h. Subsequently, 50 μL of MTT (1.0 mg/mL buffered saline phosphate) was added. After 4 h of incubation at 37 °C, the MTT culture medium was removed and replaced with 150 μL DMSO to dissolve the formazan crystals. Plates were incubated in the dark with agitation for 2 h. Optical density was determined at 540 nm using a microplate reader. All experiments were conducted with three independent trials, each with six replicas.

### 4.7. Statistical Analysis

In the cell viability results, the statistical significance between the control group and the treatments with the alkaloid fractions was evaluated by a one-way analysis of variance (ANOVA) followed by the Dunnett multiple-comparison test using the GraphPad Prism 5.0 data analysis system. The results are shown as the mean ± SD. All the experiments were carried out in triplicate.

## 5. Conclusions

In summary, this study provides relevant information about the histochemical localization of alkaloids of Amaryllidaceae in the roots and in the basal edge of scale-like leaves and bud apical zone of *P. lehmannii*. Histochemical analysis of plants grown under nursery conditions of *P. lehmannii* showed that a possible site of alkaloid biosynthesis is the basal edge of scale-like leaves and the apical zone of buds. Lycorine-type alkaloids, such as anhydrolycorine, show prevalence at the root, and crinine-type alkaloids, such as 8-*O*-demethylmaritidine, are common in the bulbs. Therefore, the roots of *P. lehmannii* represent a good source of lycorine. The bulbs constitute a significant part of the plant biomass and contain high levels of crinine-type alkaloids not detected in the root, so they could also be considered a valuable source of alkaloids. The cytotoxic potential against lung cancer cells of the root alkaloids was reported and mainly attributed to the presence of lycorine-type alkaloids. Consequently, the underground organs (bulb and root) of *P. lehmannii* are a source of alkaloids with cytotoxic potential, with interesting pharmacological properties in lung and gastric cancer.

## Figures and Tables

**Table 1:** Amaryllidaceae alkaloids identified in *Phaedranassa lehmannii* by GC–MS.

Alkaloids	RI ^1^	M^+^	m/z (Relative Intensity %)	Bulb		Root	
				% Relative ^2^	µg/g DW ^3^	% Relative ^2^	µg/g DW ^3^
**Narciclasine type**				**12.95**	**3.75**	**4.23**	**13.74**
Trisphaeridine	2235	223	223(100), 222(40), 167(12), 164(14), 138(21), 111(12).	12.95	3.75	3.49	11.33
5,6-Dihydrobicolorine	2250	239	239(15), 238(100), 180(8), 139(3).	nd	nd	0.74	2.41
**Galanthamine type**				**27.76**	**8.04**	**22.27**	**72.35**
Galanthamine	2316	287	287(86), 286(100), 244(26), 216(33), 174(31), 128(15), 115(18).	8.53	2.47	11.58	37.63
Sanguinine	2348	273	273(100), 272(74), 256(19), 212(12), 202(33), 160(43).	13.12	3.80	10.69	34.72
Epigalanthamine	2403	287	287(100), 286(86), 270(23), 244(18), 230(23), 216(29), 174(39), 128(21), 115(22).	6.11	1.77	nd	nd
**Crinane type**				**33.83**	**9.80**		
Crinine	2388	271	271(100), 270(18), 228(24), 200(32), 199(73), 187(70), 173(24), 129(32), 128(31), 115(37), 56(36).	3.56	1.03	nd	nd
8-*O*-Demethylmaritidine	2416	273	273(100), 230(23), 202(26), 201(91), 189(57), 175(24), 174(18), 129(17), 128(19), 115(20), 56(22).	30.27	8.77	nd	nd
**Haemanthamine type**						**4.17**	**13.55**
Hamayne	2620	258	287(10), 258(100), 242(14), 212(15), 211(18), 186(17), 181(18), 153(11), 128(16).	nd	nd	4.17	13.55
**Lycorine type**				**25.48**	**7.38**	**69.33**	**225.29**
Anhydrolycorine	2405	251	251(45), 250(100), 220(2), 192(12), 191(11), 165(3), 124(7).	nd	nd	41.15	133.71
11,12-Dehydroanhydrolycorine	2519	249	249(60), 248(100), 190(23), 163(8), 123(7), 95(18).	nd	nd	3.95	12.83
Lycorine	2654	287	287(25), 268(20), 250(12), 227(64), 226(100), 211(4), 147(10).	23.44	6.79	22.76	73.97
Sternbergine	2695	331	331(29), 270(26), 229(60), 228(100).	2.04	0.59	1.47	4.78

^1^ RI: Kovats retention index. ^2^ Percentages of relative peak area of compounds in the samples analyzed. ^3^ Quantitative values obtained by response factor using codeine as internal standard (µg of alkaloid per g of dry weight). nd: not detected.

**Table 2:** Cytotoxic potential of alkaloids *P. lehmannii*.

	IC_50_ (µg/mL) ± SD ^1^				
	A549 ^2^	SI ^4^	AGS ^2^	SI ^4^	HaCat ^3^
Bulb	11.76 ± 0.99	4.39	8.00 ± 1.35	6.45	51.59 ± 7.98
Root	2.59 ± 0.56	15.29	18.74 ± 1.99	2.11	39.60 ± 7.86
Lycorine ^5^	4.97 ± 0.89	7.92	4.07 ± 0.21	9.68	39.39 ± 0.94
Doxorubicin ^5^	5.52 ± 0.21	0.81	5.84 ± 0.65	0.77	4.48 ± 0.45

^1^ Results are the mean values ± standard deviations of three independent replications. ^2^ A549: lung cancer cell line and AGS: gastric cancer cell line. ^3^ HaCat: Human keratinocytes were used as control cells. ^4^ SI refers to the selectivity index (SI). ^5^ Positive controls.

## Data Availability

The raw data supporting the conclusions of this article will be made available by the researchers upon request.
